# The underestimated role of plant root nitric oxide emission under low-oxygen stress

**DOI:** 10.3389/fpls.2024.1290700

**Published:** 2024-02-06

**Authors:** Marcel Welle, Wiebke Niether, Christine Stöhr

**Affiliations:** Plant Physiology, Institute of Botany and Landscape Ecology, University of Greifswald, Greifswald, Germany

**Keywords:** nitric oxide emissions, root, oxygen deprivation, hypoxia, low oxygen stress

## Abstract

The biotic release of nitric oxide (NO), a greenhouse gas, into the atmosphere contributes to climate change. In plants, NO plays a significant role in metabolic and signaling processes. However, little attention has been paid to the plant-borne portion of global NO emissions. Owing to the growing significance of global flooding events caused by climate change, the extent of plant NO emissions has been assessed under low-oxygen conditions for the roots of intact plants. Each examined plant species (tomato, tobacco, and barley) exhibited NO emissions in a highly oxygen-dependent manner. The transfer of data obtained under laboratory conditions to the global area of farmland was used to estimate possible plant NO contribution to greenhouse gas budgets. Plant-derived and stress-induced NO emissions were estimated to account for the equivalent of 1 to 9% of global annual NO emissions from agricultural land. Because several stressors induce NO formation in plants, the actual impact may be even higher.

## 1 Introduction

Climate change causes elevated temperatures, enhanced drought periods, and heavy rainfall and affects all life forms on Earth (Trenberth, 2005). In particular, plants are subjected to increasingly extreme weather conditions because of their sessile lifestyles and are forced to endure stressors with all their consequences (Prasch and Sonnewald, 2015). The impact and response of plants to these stressors have been extensively discussed and investigated from different perspectives (Bigot et al., 2018; Cassia et al., 2018; Agnihotri and Mitra, 2023). However, the influence of stressed plants on climate change remains unclear.

The factors that predominantly influence the climate are the quantity and composition of greenhouse gases. Trace gases in the atmosphere have complex chemistry and may form atmospheric oxidants such as ozone and hydroxyl radicals. Nitric oxide (NO) contributes significantly to this production (Pilegaard, 2013). Interestingly, nitric oxide can also be microbially reduced to nitrous oxide (N_2_O) (Cameron et al., 2013; Caranto and Lancaster, 2017), a greenhouse gas with 265-298 times higher global warming potential than that of carbon dioxide (IPCC, 2014). One major source of nitric oxide is the soil of agricultural and forest systems (Davidson and Kingerlee, 1997; Stehfest and Bouwman, 2006). Soil NO emissions have been dependent on many factors, such as soil water content, soil temperature, soil pH, ambient NO concentration, soil organic carbon, and nitrogen availability (Ludwig et al., 2001; Pilegaard, 2013). Among the processes of soil NO formation, microbial nitrification and denitrification are considered the major processes (Pilegaard, 2013), whereas abiotic processes are suggested to play a minor role only under low pH conditions. The contribution of soil-derived NO to the total annual tropospheric NO budget has been estimated to be as high as 8% for Saxony (Molina-Herrera et al., 2017).

However, little attention has been paid to the contribution of higher plants to the global NO budget. In plants, NO functions as a signaling molecule and has been well characterized in recent years (Astier et al., 2018). NO plays a crucial role in plant development, including germination, flowering, and leaf senescence, as well as in adaptation to biotic and abiotic stress (reviewed by Nabi et al., 2019). Especially in roots, NO signaling controls several processes in organogenesis (Pagnussat et al., 2002). Under stress conditions, NO induces aerenchyma and adventitious root formation (Chen et al., 2016; Wany et al., 2017).

An increase in plant NO emissions in abiotic stress responses has been described for many stressors such as salt, drought, heavy metals (reviewed by Nabi et al., 2019), and hypoxia (Gupta et al., 2005; Stöhr and Stremlau, 2006; Safavi-Rizi et al., 2020). Hypoxic conditions in terrestrial plants occur primarily after flooding and heavy rainfall (Bailey-Serres et al., 2012). Under these conditions, NO emitted from the soil is thought to be produced through the microbial denitrification process (Ludwig et al., 2001). However, hypoxic conditions in the soil may also lead to a significant increase in NO formation in plant root cells (Gupta et al., 2005; Stöhr and Stremlau, 2006; Liu et al., 2015). A reductive pathway for NO formation triggered by oxygen deprivation has been suggested (Timilsina et al., 2022). First, nitrate is reduced to nitrite by nitrate reductase (Igamberdiev and Hill, 2004). Subsequently, nitrite is thought to be reduced to NO. Among the possible NO-forming enzymes in plants, cytosolic nitrate reductase (Rockel et al., 2002; Meyer et al., 2005), PM-bound nitrite:NO reductase (Stöhr et al., 2001; Stöhr and Ullrich, 2002; Stöhr and Stremlau, 2006), and mitochondrial electron transport chain (mETC) complexes III and IV (Gupta et al., 2005; Gupta and Igamberdiev, 2011) induce NO formation under low-oxygen conditions. Furthermore, NO-forming enzymes containing molybdenum cofactors have also been identified (Wang et al., 2010; Chamizo-Ampudia et al., 2016; Chamizo-Ampudia et al., 2017; Astier et al., 2017). The NO-forming activity of most of these enzymes is assumed to depend on oxygen. The reductive NO formation pathway is thought to fight energy shortages in oxygen-deprived plants, and NO acts as a final electron acceptor (Igamberdiev and Hill, 2004). The extent to which low-oxygen-induced NO emissions contribute to annual global NO emissions is unknown.

In this study, low-oxygen-induced NO emissions were investigated by measuring the entire root system of intact plants (*in vivo*) of different species (tomato, tobacco, and barley). Protoplasts (*in situ*) and aseptically cultured plants (*in vivo*) were assessed to clarify the origin of NO emission. Based on the obtained data, the contribution of plant-derived NO to the global NO budget during low-oxygen periods was estimated.

## 2 Plant roots emit NO in an oxygen-dependent manner

**Figure 1:**
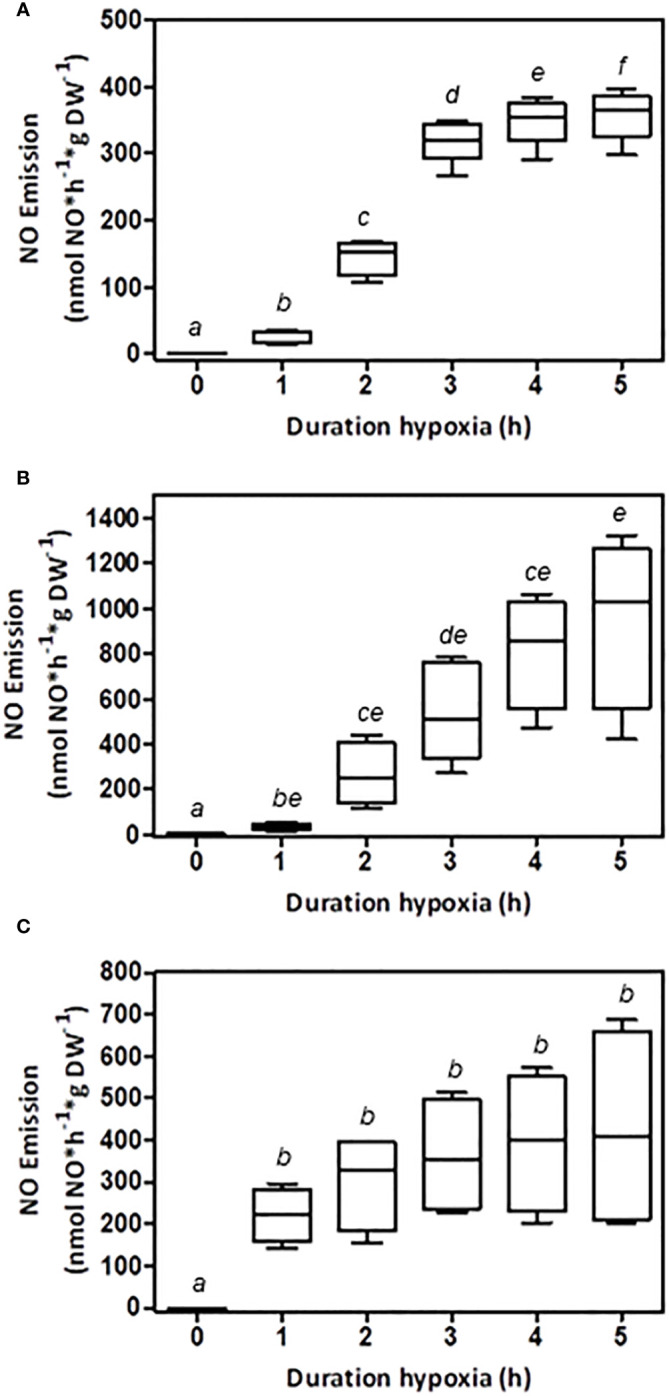
Effect of oxygen on NO emission from root systems of different plant species. Hydroponically grown plants were transferred to the root reactor, and NO emissions were measured *in vivo*. The shoot was exposed to ambient air while the root was humidified. An airflow of nitrogen gas was used to achieve low-oxygen conditions inside the sealed root reactor. NO emissions were measured by chemiluminescence. Shown are the values after 0, 1, 2, 3, 4, and 5 hours under low-oxygen conditions. **(A)** NO emissions from tobacco roots (n=6), **(B)** NO emissions from tomato roots (n=5), and **(C)** NO emissions from barley roots (n=5). Boxplots are shown and plotted with median and SD. The significance was tested by multiple paired t-tests with P ≤ 0.05. Boxes with the same letter are not significantly different.

The determination of plant NO emissions *in vivo* without impairing organs is an important tool for assessing and evaluating the impact of stress on plants and climate. In this study, the *in vivo* NO emissions of three plant species under low-oxygen conditions were examined. Plant root NO emissions were measured for up to 5 h in intact plants that experienced sufficient oxygen during their growth period (**Figure 1**). After the initial adjustment to low-oxygen conditions in the reactor, a strong increase in NO emissions was observed. All investigated plant species attained a constant rate of NO formation after 3–5 h, depending on the species. Tobacco roots emitted a maximum of approximately 365 nmol NO*g DW^-1^*h^-1^ (**Figure 1A**), while tomato roots produced up to 1,028 nmol NO*g DW^-1^*h^-1^ (**Figure 1B**). The barley root NO release averaged 407 nmol NO*g DW^-1^*h^-1^ (**Figure 1C**). In addition to the maximum NO emissions, the initial adaptation phase was specific to each species. While barley showed rapid adjustment within 2 h, tobacco required approximately 3 h, and tomato plants required 4 h to attain a constant NO emission rate. All investigated plant species revealed low but determinable NO emissions at the beginning of the measurement, reflecting the state of former normoxia with an explicit increase in response to low oxygen over time.

**Figure 2:**
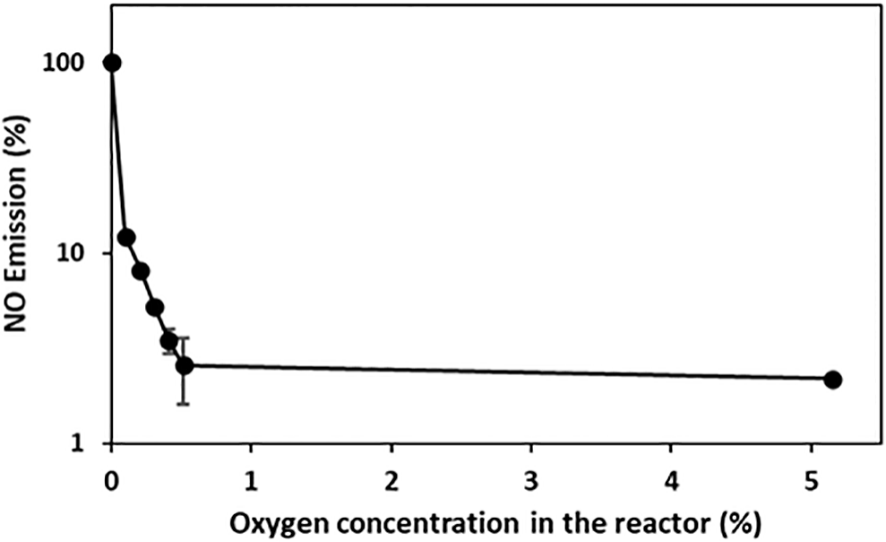
Dependence of NO emissions on oxygen concentration. *In vivo* NO emission was recorded from tobacco roots exposed to an airstream with different oxygen concentrations (0, 0.103, 0.206, 0.309, 0.412, 0.515, 5.150% oxygen). The mean and SD (n=3) are shown. The NO emissions are shown on a logarithmic scale.

To investigate the impact of oxygen on NO emissions, the roots of intact tobacco plants were exposed to gas flow at various oxygen concentrations. Only a minor amount of NO was emitted from the tobacco roots at 5.15% oxygen in the nitrogen gas. An increase in NO emissions was observed at oxygen concentrations of < 0.515%. The root systems produced the highest NO emissions under anoxic conditions. A correlation between the oxygen level and relative NO emissions was observed (**Figure 2**).

## 3 Plants contribute to global NO emissions under low-oxygen conditions

The sources of NO emissions from agricultural systems are usually soil-borne microbes (Pilegaard, 2013; Yu and Elliott, 2021). However, low-oxygen-induced NO emissions appear to be a general adaptation in higher plants when nitrate is abundant (**Figure 1**; Rockel et al., 2002; Gupta et al., 2005; Hebelstrup et al., 2012; Mugnai et al., 2012; Oliveira et al., 2013; Liu et al., 2015). The proportion of stress-induced NO emissions by plants to the global annual NO emissions remains elusive.

To facilitate the comparison of data obtained under controlled conditions with field NO emissions, the determined NO emission rates were estimated for the annual NO produced per hectare for each of the investigated plant species.

The following assumptions were made:

1. Root DW of single mature plants in the field was taken from literature for tobacco [Wolf and Bates (1964)], tomato [Brdar-Jokanović et al. (2014)], and barley (Heydari et al. (2019).
2. Values for plant density per ha were based on the work of Reynolds et al. (2022) for tobacco, Elattir (2002) for tomato, and Paynter and Hills (2009) for barley.
3. The average duration of a flooding period was assumed to be 12 days according to Pendergrass and Knutti (2018), who found half of annual precipitation falls within the wettest 12 days worldwide. The occurrence of low-oxygen conditions by submergence due to heavy rainfalls or flooding was therefore assumed to be within this time period.
4. Flooding results in either anoxic or hypoxic soil conditions that are dependent on soil type and composition and oxygen consumption in the soil (Ponnamperuma, 1972). Based on **Figure 2**, hypoxic conditions may account for 10% of anoxic NO emissions.
5. The detected NO emission rate after 5 h of low-oxygen conditions (**Figure 1**) was extrapolated for the entire assumed flooding period of 1 year.

Based on these assumptions, the annual NO emissions per hectare planted with different plant species were calculated. Under low-oxygen soil conditions, tobacco roots emitted the lowest NO amount per hectare, followed by tomatoes and barley (**Table 1**). Under anoxic conditions, the NO emission of each species was 10 times higher than that under hypoxic conditions. In addition to oxygen availability, the main factor that determines NO emissions per hectare is plant density, which depends on the plant size.

**Table 1:** Estimation of oxygen-depleted NO emissions per hectare and year from three different plant species based on reactor *in vivo* data.

	Tobacco	Tomato	Barley	Reference
**Plant NO emission** (nmol NO*h^-1^*g DW ^-1^)	365	1028	407	**Figure 1**
**Root dry weight** (g DW*plant^-1^)	25 ^a^	6.7 ^b^	1.5 ^c^	^a^ Wolf and Bates (1964) ^b^ Brdar-Jokanović et al. (2014) ^c^ Heydari et al. (2019)
**Annual flooding period** (h*a^-1^)	288 ^g^	288 ^g^	288 ^g^	^g^ Pendergrass and Knutti (2018)
**Planting density** (plant*ha^-1^)	15000 ^d^	30000 ^e^	2000000 ^f^	^d^ Reynolds et al. (2022) ^e^ Elattir (2002) ^f^ Paynter and Hills (2009)
**Annual NO emission – anoxic soil conditions** *(*g NO-N*ha^-1^*a^-1^ *)*	552	833	4,917	–
**NO emissions per ha – hypoxic soil conditions (10%)** *(*g NO-N*ha^-1^*a^-1^ *)*	55	83	492	Based on **Figure 2**

The annual NO emission was calculated as: *Annual NO emission = Plant NO emission ∗ root dry weight ∗ annual flooding period ∗ planting density ∗ molecular weight NO/molecular weight nitrogen*. For the NO emissions of the plants, median values of the NO emission data after 5 h shown in **Figure 1** were used. **Figure 2** shows that residual oxygen in the soil leads to 10% of the anoxic NO emissions.

## 4 Discussion

Fossil fuel combustion and microbial emissions are considered to be the main sources of NO emissions into the atmosphere (Pilegaard, 2013). The impact of plants on NO emissions has rarely been acknowledged, although the formation of NO by plants has been known for more than 40 years (Kolbert et al., 2019). Low-oxygen-induced NO formation in plants has been found in a diversity of species such as sunflower (detached leaf) (Rockel et al., 2002), *Arabidopsis thaliana* (rosettes) (Hebelstrup et al., 2012), barley, pea, tobacco (root segments and extracts) (Gupta et al., 2005), poplar (*root, stem, and leaf*) (Liu et al., 2015), soybean (root) (Oliveira et al., 2013), and maize (seedling root) (Mugnai et al., 2012). In this study, *in vivo* NO emissions from the root system under low-oxygen conditions were measured in three different plant species. Plants were treated as unaffected as possible to minimize the effects of additional stressors. For all these, a reproducible and similar NO emission course was determined. Based on plant NO emissions, the impact of low-oxygen-stressed plants on global annual NO emissions from agricultural land was assessed. According to our estimation, between one and nine percent of annual NO emissions may be caused by plants under low-oxygen stress.

To ascertain the plant origin of NO formation and exclude microbial contributions, as highlighted by Horchani et al. (2011), root protoplasts and aseptically grown plants were measured. Protoplast isolation can reduce microbial contamination by several washing steps and structural dissolution. NO formation by protoplasts in response to low-oxygen conditions resembled NO emission in hydroponically grown plants (**Supplementary Figure 1A**). Although the NO emissions from aseptically grown plants were much lower than those from hydroponically grown plants, they also responded to low-oxygen conditions (**Supplementary Figure 1B**). Differences in NO formation between aseptic- and non-aseptic-cultured plants may be due to the culture conditions on agar medium versus hydroponic culture, as indicated by the reduced growth rate (data not shown). Potential growth limitations owing to a shortage of nutrients, particularly nitrogen, may cause low NO formation under these conditions (Stöhr and Stremlau, 2006; Liu et al., 2015). Overall, there is strong evidence that oxygen-dependent NO formation is directly derived from plant root systems. The detected *in vivo* NO emissions by plant root systems support the results of previous studies. The correlation between low oxygen levels and increased NO emissions has been extensively studied in various plant segments and extracts (Rockel et al., 2002; Gupta et al., 2005; Meyer et al., 2005; Stöhr and Stremlau, 2006). Therefore, NO emissions are highly dependent on oxygen. Anoxic conditions result in the highest NO emissions (Gupta et al., 2005; Stöhr and Stremlau, 2006). A total of 50% of the total NO formation in the plant extracts (I_50_) was observed at oxygen concentrations between 5% (plasma membrane related: Stöhr and Stremlau, 2006) and 0.05% (mitochondria related: Gupta et al., 2005). The measured I_50_ of *in vivo* root NO emissions was equal to that of mitochondria-derived NO emissions. This is consistent with the view that mitochondria are one of the main sources of NO in response to low-oxygen conditions (reviewed by Gupta and Igamberdiev, 2011).

In accordance with *in vivo* measurements of poplar roots (Liu et al., 2015), NO emissions increased shortly after the onset of low-oxygen conditions. Maximum NO emission was observed after 3–5 h (**Figure 1**). It is possible that the effect of residual oxygen levels in the root tissue on NO formation must be considered. The maximum NO emissions related to fresh weight ranged from 65 to 20 nmol NO g FW^-1^ h^-1^ for tomatoes, tobacco, and barley (**Figure 1**). These values were comparable to those recorded in hypoxia-treated soybean roots (60 nmol NO g FW^-1^ h^-1^; Oliveira et al., 2013). In flood-tolerant poplar species, however, hypoxic conditions resulted in root NO emission of 0.05 nmol NO g FW^-1^ h^-1^ (Liu et al., 2015). It has been speculated that flood-tolerant species show lower hypoxic root NO formation than sensitive species (Liu et al., 2015). These results supported our hypotheses. However, most crop plants are suggested to respond sensitively to flooding stress (reviewed by Mustroph, 2018). In addition to the tested species (tomato, tobacco, and barley), other important crop plants have shown to undergo induction of NO emissions upon oxygen deprivation. Plant materials of maize (Mugnai et al., 2012) and peas (Gupta et al., 2005) showed similar low-oxygen NO formation, which further supports the idea of a ubiquitous plant response. This assumption needs to be confirmed in a range of species and genotypes in future experiments.

Global annual NO emissions from fertilized crops and grasslands were assumed to be 1.8 Tg NO-N (Stehfest and Bouwman, 2006). Yan et al. (2015) measured the NO emissions in cereal fields under different management regimes within the same range. Soil NO emissions have been observed to depend on many factors, including soil water content, soil temperature, and nitrogen availability (Pilegaard, 2013; Ludwig et al., 2001). However, most agriculturally related NO emissions are linked to nitrogen fertilization (Li and Wang, 2008). Among the processes of soil NO formation, microbial nitrification and denitrification are considered the major processes (Pilegaard, 2013). Little attention has been paid to abiotic processes and plants themselves as potential sources of NO. The global area contributing to NO emissions is assumed to be 1906 Mha, including croplands and grasslands (Stehfest and Bouwman, 2006). This amounts to 944 g of NO-N per hectare per year.

To assess an order of magnitude of plant-derived, hypoxia-induced NO emissions, the NO formation rates obtained from laboratory experiments were expanded to the field. Under the assumption that all crop plants show a similar range of low-oxygen-induced NO emissions, NO emitted per ha per year may account for 552–4917 g depending on species and planting density (**Figure 1**). A critical parameter for the extrapolation of plant NO emissions under low-oxygen stress appears to be the oxygen content of the submerged soils (**Figure 2**). The rate of oxygen depletion in soil is dependent on soil structure and composition (Ponnamperuma, 1972). The presence of microbes and plants is critical for consuming remaining oxygen (Pradet and Bomsel, 1978). Reports on the development of anoxic soil conditions as a consequence of flooding range from a few hours (Ponnamperuma, 1972) to days (Scott and Evans, 1955). A recent study identified permanent anoxic microsites in non-flooded soils (Keiluweit et al., 2018). Further increases in the amount of heavy rainfall and flooding periods due to climate change (Tabari, 2020) may increase the frequency of anoxic soil conditions, thereby intensifying the impact of stressed plants on annual NO emissions. With 12 days a year and half of the annual precipitation (Pendergrass and Knutti, 2018), the assumption of low-oxygen conditions in the soil during those days is most likely. Reports on NO transport and storage within plants (Liu et al., 2015; Manrique-Gil et al., 2021), NO uptake by the plant canopy (Bennett and Hill, 1973), and possible adaptations (Wany et al., 2017) add an additional layer of complexity to estimating the impact of plant NO emissions on global NO emissions. The applied method excluded some natural uncertainties such as soil structures and diverse nutrient compositions; however, more species need to be tested to obtain a better picture. Therefore, an understated estimation of plant NO emissions based on the observed hypoxic conditions (0.103% oxygen, **Figure 2**) may be more realistic. Several other stressors, including drought, salinity, and heavy metal stress (reviewed by Nabi et al., 2019), may also increase NO emissions by plants. However, the impact on global NO emissions may become increasingly important with an increase in extreme weather events.

NO has the potential to be converted into N_2_O, a strong greenhouse gas (Cameron et al., 2013; IPCC, 2014; Caranto and Lancaster, 2017). Recent studies have identified plants as producers of N_2_O via a pathway involving an NO intermediate under low-oxygen stress. The exact pathways and involvement of soil and plants remain elusive. A protective function to reduce NO concentrations was discussed by Timilsina et al. (2020). Application of nitrogen isotope-tagged NO to soil and plant settings may help reveal the N_2_O conversion mechanism and the involved fractions. NO measurements in the field are primarily performed in chambers spanning the aboveground soil and plants (Rothardt et al., 2021). Various farmland crops have been cultivated using this technique (reviewed by Pan et al., 2022). Microbial denitrification emissions have been widely studied. The lack of separate measurements of plant- and soil-derived emissions likely masks the impact of plant NO emissions on global NO emissions. This makes it difficult to evaluate the effect of plants on global NO emissions. Machacova et al. (2019) developed a separate chamber setting for the measurement of N_2_O emissions from trees. To date, no fitting system for crop plants has been developed. Isotopic fingerprints for NO sources, similar to those described for N_2_O emissions (Decock and Six, 2013), could be an option. An additional challenge is the application of abiotic stressors such as flooding to a chamber-based measurement system. Separate plant feeding and transferring to the field could overcome these limitations.

## 5 Methods

*Solanum lycopersicum* (L.) cv. Moneymaker was grown under greenhouse conditions (14 h light 18°C/10 h darkness 22°C). The tomato plants were grown for 2 weeks on a quartz sand culture with a nutrient solution containing 5 mM NO_3_^-^ (Stöhr and Ullrich, 1997; Wendlandt et al., 2016). The tomato plants were then grown for 5 weeks in a hydroponic culture with the same nutrient solution. The nutrient solution was replaced every 3 days. The hydroponic roots were aerated with ambient air using an ACO-9620 (Hailea) aquarium air pump and an AS 25 air diffuser (Tetra Tec). *Nicotiana tabacum* (L.) cv. Samsun seeds were germinated on filter paper soaked with CaSO_4_ solution at room temperature and grown for 2 weeks on a quartz sand culture with a nutrient solution containing 5 mM NO_3_^-^ (Stöhr and Ullrich, 1997; Wendlandt et al., 2016) before being transferred to the same growth regime as described for the tomato plants. *Hordeum vulgare* (L.) cv. seeds were soaked in water. Afterward, the seeds were then sterilized in a 0.5% sodium hypochlorite solution. The seeds were germinated for 8 days on filter paper soaked in CaSO_4_ solution. The CaSO_4_ solution was replaced with a nutrient solution containing 5 mM NO_3_^-^ (Stöhr and Ullrich, 1997; Wendlandt et al., 2016), and the plants were grown for 7 days. The conditions in the greenhouse corresponded to those described for the tomato plants. For aseptic growth conditions, *Nicotiana tabacum* (L.) cv. Samsun seeds were sterilized in 70% ethanol, followed by a 2% sodium hypochlorite solution containing 0.1% Tween 20. Seeds were germinated on plates containing 1.4% (w/v) bacto-agar, 0.5 mM calcium sulfate, and 2% (w/v) sucrose. Plants were transferred to a 50 ml Erlenmeyer flask containing McCown woody plant medium (Lloyd and McCown, 1980). Plants were grown for 3 months in the climate chamber (14 h light (28°C) and 10 h darkness (22°C).

Intact plants were transferred to a custom-built airtight and opaque reactor (4 l, LMS GmbH Ilmenau). Roots were placed into the reactor while the shoot was exposed to light and ambient air. The reactor was attached to a thermostat (Thermo Electron Corporation, 25°C) and an irrigation system M.R.S Micro Nebeldüse (Micro Rainfall Systems) with a piston pump V1 (Micro Rainfall Systems) to avoid desiccation of the roots. Possible leaks were sealed with Optosil (Heraeus). NO was measured with the chemiluminescence-based NO detector system ANALYZER LCD 88 sp (Eco Physics) with two cold traps [glass bottle on ice (4°C), REFRIGERATED APOR TRAP RVT 100 (Savant) (-50°C)], and a gas hose heater (custom-made, workshop University of Greifswald) upstream of the detector. Humidification of the N_2_ carrier gas at a flow rate of 400 mL min^-1^ was ensured using ultrapure water in a fritted gas-washing bottle gas as described by Stöhr and Stremlau (2006) and depicted in **Supplementary Figure 2**. The oxygen concentration was adjusted by mixing compressed air (air–liquid) and N_2_ gas. The system was calibrated with 86 ppb NO in N_2_ gas and adapted to the NO analyzer in accordance with the manufactory guidelines. The addition of a mixture of NO gas and compressed air assured correct NO detection in the presence of O_2_. The detected NO concentrations were in accordance with the calculated ones. The baseline was recorded with the empty reactor. The NO production was calculated relative to the root dry weight.

## Data availability statement

The original contributions presented in the study are included in the article/**Supplementary Material**. Further inquiries can be directed to the corresponding author.

## Author contributions

MW: Conceptualization, Investigation, Writing – original draft. WN: Investigation, Writing – review & editing. CS: Conceptualization, Supervision, Writing – review & editing.

## Supplementary material

Supplementary Figure 1 Root protoplasts and aseptically grown plant roots emit NO under low-oxygen conditions. **(A)** NO emission from protoplasts isolated from 3-week-old roots of hydroponically grown tomato plants was measured *in situ*. Shown are the values after 0, 1, 2, 3, 4, and 5 h under low-oxygen conditions. Mean and SD (n=8) are shown. **(B)** *In vivo* aseptically grown tobacco plants. Shown are the values after 0, 1, 2, 3, 4, and 5 hours under low-oxygen conditions. Shown are the mean and SD (n=3).

Supplementary Figure 2 Chemiluminescence-based NO detection setup. The reactor allowed the integration of the whole plant root system (4 l reactor) into the NO detection system. Humidification of the carrier gas (N_2_ or O_2_) and irrigation ensured the viability of the sample. While the shoot was lightened, the dark root reactor was temperature controlled. NO was transported to the chemiluminescence detector via a constant gas flow (direction shown by black arrows). Two cold traps and a gas hose heater were installed prior to the NO chemiluminescence detector to protect it.
