# Supplementary figures and images for: The underestimated role of plant root nitric oxide emission under low-oxygen stress
Source: Front Plant Sci. 2024 Feb 6;15:1290700. doi: 10.3389/fpls.2024.1290700 (PMC10876902; doi:10.3389/fpls.2024.1290700)

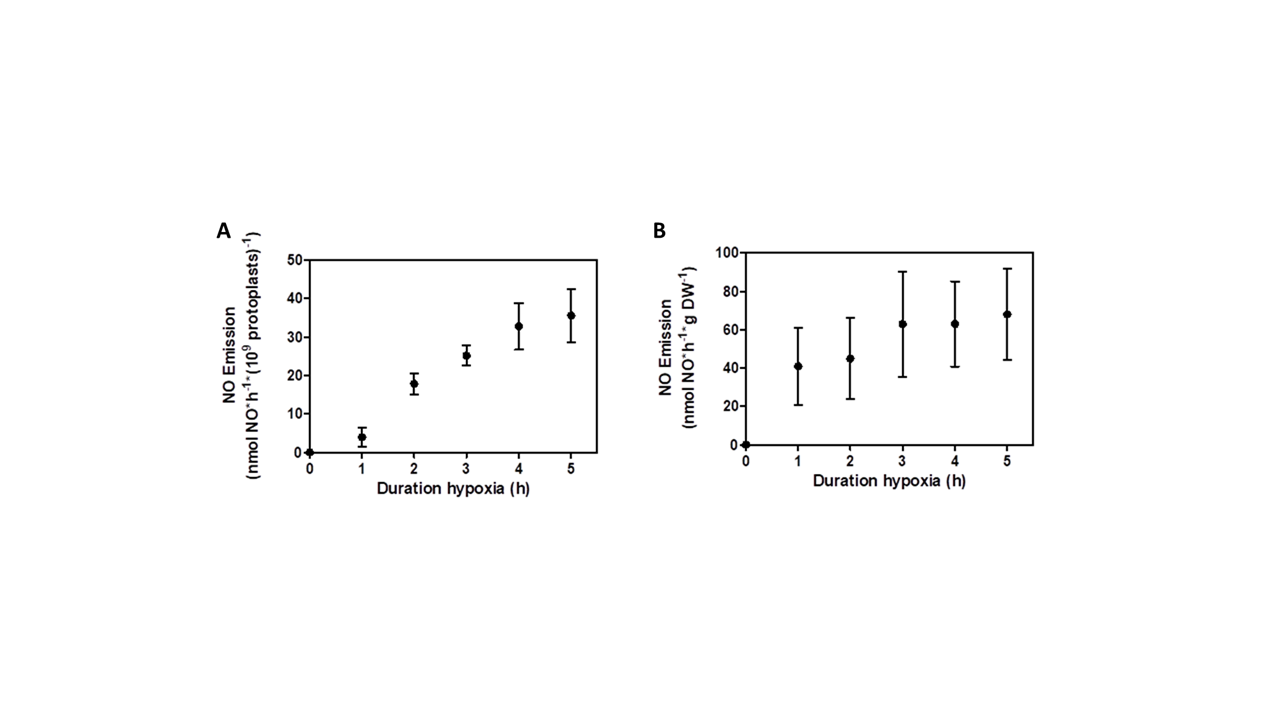

Supplement: Supplementary Figure 1 — Root protoplasts and aseptically grown plant roots emit NO under low-oxygen conditions. (A) NO emission from protoplasts isolated from 3-week-old roots of hydroponically grown tomato plants was measured in situ. Shown are the values after 0, 1, 2, 3, 4, and 5 h under low-oxygen conditions. Mean and SD (n=8) are shown. (B) In vivo aseptically grown tobacco plants. Shown are the values after 0, 1, 2, 3, 4, and 5 hours under low-oxygen conditions. Shown are the mean and SD (n=3). [file Image_1.tif]

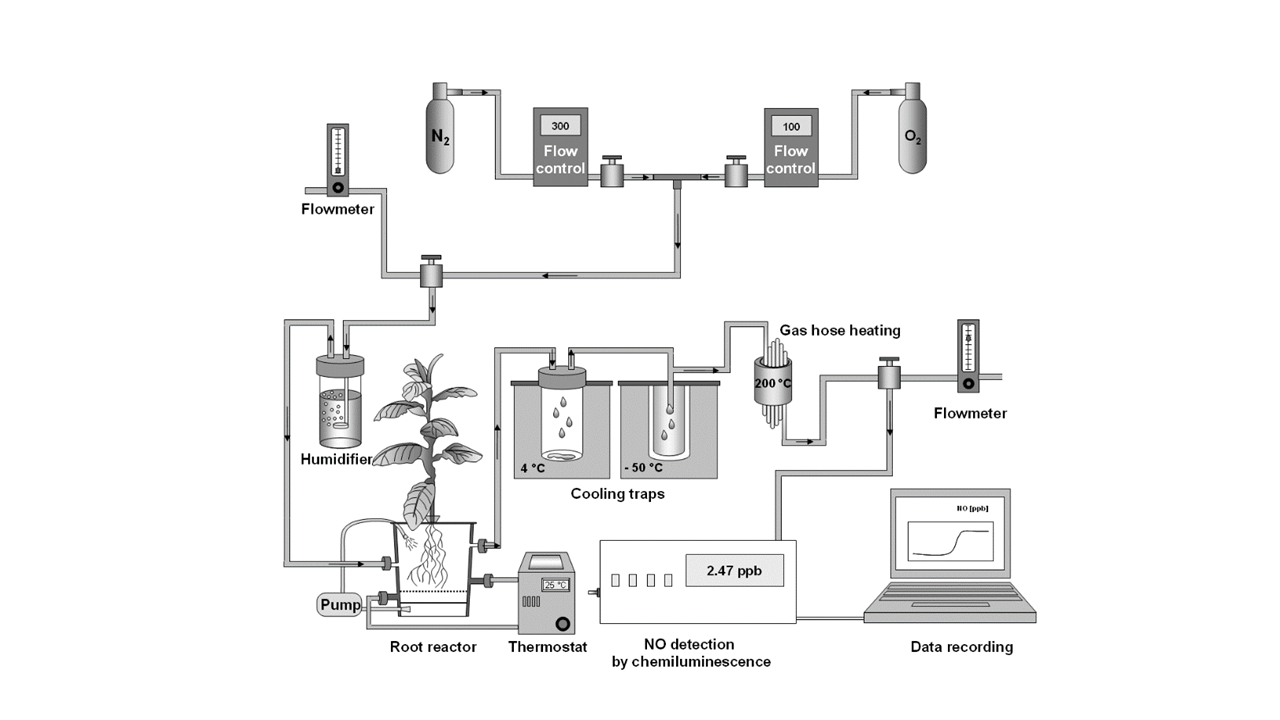

Supplement: Supplementary Figure 2 — Chemiluminescence-based NO detection setup. The reactor allowed the integration of the whole plant root system (4 l reactor) into the NO detection system. Humidification of the carrier gas (N2 or O2) and irrigation ensured the viability of the sample. While the shoot was lightened, the dark root reactor was temperature controlled. NO was transported to the chemiluminescence detector via a constant gas flow (direction shown by black arrows). Two cold traps and a gas hose heater were installed prior to the NO chemiluminescence detector to protect it. [file Image_2.tif]
